# Reference intervals of gestational sac, yolk sac, embryonic length, embryonic heart rate at 6–10 weeks after in vitro fertilization-embryo transfer

**DOI:** 10.1186/s12884-020-03186-2

**Published:** 2020-09-14

**Authors:** Yan Ouyang, Jiabi Qin, Ge Lin, Shuanglin Xiang, Xihong Li

**Affiliations:** 1 College of Life Science, Hunan Normal University, Changsha, China; 2 Reproductive and Genetic Hospital of CITIC-Xiangya, Changsha, China; 3 Clinical Research Center For Reproduction and Genetics in Hunan Province, Changsha, China; 4 Department of Epidemiology and Health Statistics, Xiangya School of Public Health, Central South University, Changsha, China

**Keywords:** Reference interval, Crown-rump length, Embryonic heart rate, Gestational sac, Yolk sac, In vitro fertilization-embryo transfer, First trimester

## Abstract

**Background:**

Accurately determining the normal range of early pregnancy markers can help to predict adverse pregnancy outcomes. The variance in ovulation days leads to uncertain accuracy of reference intervals for natural pregnancies. While the gestational age (GA) is accurate estimation during in vitro fertilization-embryo transfer (IVF-ET). Thus, the objective of this research is to construct reference intervals for gestational sac diameter (GSD), yolk sac diameter (YSD), embryonic length (or crown–rump length, CRL) and embryonic heart rate (HR) at 6–10 gestational weeks (GW) after IVF-ET.

**Methods:**

From January 2010 to December 2016, 30,416 eligible singleton pregnancies were retrospectively recruited. All included participants had full records of early ultrasound measurements and phenotypically normal live neonates after 37 GW, with birth weights > the 5th percentile for gestational age. The curve-fitting method was used to screen the optimal models to predict GSD, CRL, YSD and HR based on gestational days (GD) and GW. Additionally, the percentile method was used to calculate the 5th, 50th, and 95th percentiles.

**Results:**

There were significant associations among GSD, CRL, YSD, HR and GD and GW, the models were GSD = − 29.180 + 1.070 GD (coefficient of determination [R^2^] = 0.796), CRL = − 11.960 - 0.147 GD + 0.011 GD^2^ (R^2^ = 0.976), YSD = − 2.304 + 0.184 GD - 0.011 GD^2^ (R^2^ = 0.500), HR = − 350.410 + 15.398 GD - 0.112 GD^2^ (R^2^ = 0.911); and GSD = − 29.180 + 7.492 GW (R^2^ = 0.796), CRL = − 11.960 - 1.028 GW + 0.535 GW^2^ (R^2^ = 0.976), YSD = − 2.304 + 1.288 GW - 0.054 GW^2^ (R^2^ = 0.500), HR = − 350.410 + 107.788 GW - 5.488 GW^2^ (R^2^ = 0.911), (*p* < 0.001).

**Conclusions:**

Reference intervals for GSD, YSD, HR and CRL at 6–10 gestational weeks after IVF-ET were established.

## Background

Accurately determining the normal range of early pregnancy markers can help to predict adverse pregnancy outcomes, such as miscarriage. It is also useful to determine the number of foetuses and their viability, type of twinning, and presence of gross fetal abnormalities, placental problems, and uterine or adnexal problems. Some studies have constructed reference intervals that mostly depend on natural conceptions of women with regular menstrual cycles and known dates of their last menstrual periods (LMPs) [1–3]. However, a discrepancy of more than 7 days in gestation calculated by menstrual history and by ultrasound was found in approximately 15% of women with regular menstrual cycles and specific LMP dates due to the variance in ovulation days [4]. Thus, ultrasound measurements of embryonic and foetal crown–rump length (CRL) are useful to estimate gestational age (GA) in early pregnancy [5, 6], and the classic Robinson curve is the most common method [7]. However, some researchers have shown that there is generalized underestimation of GA by the Robinson curve [8, 9]. These findings have led to uncertain accuracy of reference intervals for natural pregnancies.

During in vitro fertilization-embryo transfer (IVF-ET), the day of oocyte retrieval and ET are known; thus, the GA estimation is accurate. We speculate that reference intervals derived from IVF-ET data are more accurate than those derived from natural conception and be more suitable for IVF populations. With the rapid development of artificial reproductive technology, especially after the implementation of the two-child policy in mainland China, more infertile couples conceive with this treatment [10, 11]. However, there is no research focused on constructing reference intervals for 4 ultrasound indicators of early pregnancy following IVF-ET or specifically targeting the Chinese population.

This study analysed data from a large cohort of 30,416 singleton pregnancies with normal outcomes from a Chinese population, aiming to construct reference intervals for gestational sac diameter (GSD), yolk sac diameter (YSD), heart rate (HR) and CRL at 6–10 gestational weeks (GW) following IVF-ET. The optimal models for predicting GSD, CRL, YSD and HR based on GA were also analysed.

## Methods

### Patients

**Fig. 1:**
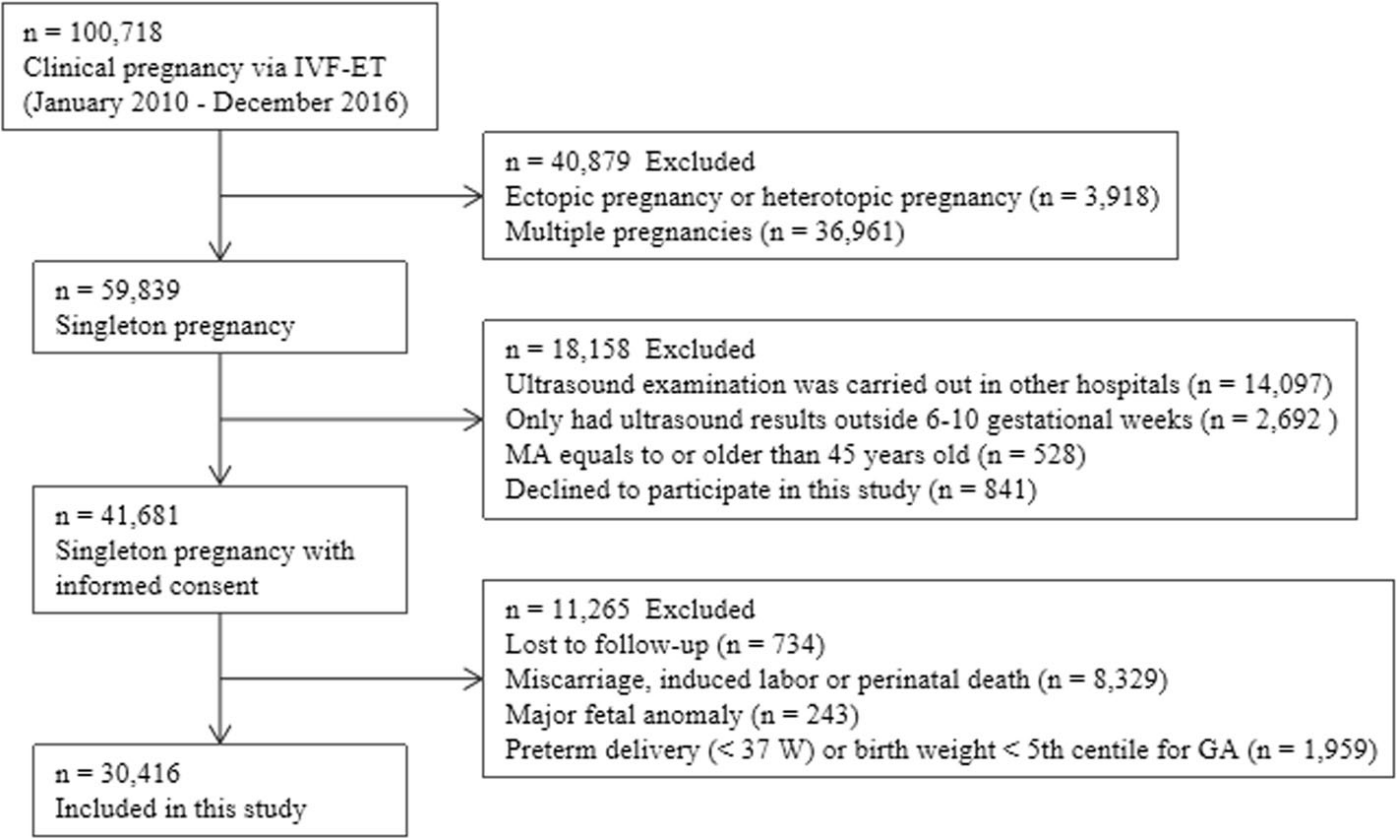
Flow chart of patient inclusion. IVF-ET, in vitro fertilization-embryo transfer; MA, maternal age; GA, gestational age

The institutional review board approved this study before data collection (LL-SC-2019-015). The study was conducted using anonimized dataset of patients for research purposes and that it was conducted in agreement with Helsinki declaration for research ethics. STROBE Guidelines were followed for reporting this observational study [12]. This retrospective study was involved 30,416 singleton pregnancies following IVF-ET at the Reproductive and Genetic Hospital of CITIC Xiangya from January 2010 to December 2016 (Changsha, China, Fig. 1). In order to create models that are applicable to more patients, the study population was minimally selected. The age of women in the studied population was up to 45 years. Due to the retrospective nature of this study, informed consent was waived. The kinds of insemination methods included IVF, intracytoplasmic sperm injection (ICSI), IVF/ICSI, and preimplantation genetic diagnosis (PGD). In these patients, 1–3 fresh or frozen embryos with good quality were transferred at the day-3 or day-5 stage, and the embryo scoring method was described in our previous studies [13, 14]. Serum-human chorionic gonadotropin (hCG) levels were measured on day 14 (blastocysts on day 12), and transvaginal scans were usually performed in the first trimester to confirm clinical pregnancy.

Pregnancy and perinatal outcomes were tracked by a specified team via telephone call or fax at our centre. The inclusion criteria were as follows: (1) live singleton pregnancy following IVF-ET; (2) embryonic GSD, YSD, HR were fully measured and recorded at 6–10 GW; and (3) live birth after 37 GW of a phenotypically normal neonate with a birth weight > 5th percentile for GA [15]. All enrolled women were informed the possibility of using their ultrasound records from the first trimester to construct reference intervals before ultrasound examinations were performed.

### Ultrasound measurements

We collected the first ultrasound examination results from each patient during 6–10 GW. The ultrasound scans were performed by 4 experienced sonographers with a GE VOLUSON E8/730 (General Electric Tech Co., Ltd., New York, USA) equipped with a 5–9 MHz transvaginal probe. The measurements referred to the ISUOG practice guidelines [16] and conformed to uniform standards: GSD was calculated the mean value of 3 perpendicular diameters with the callipers placed at the inner edges of the trophoblast; YSD was calculated as the average of 3 perpendicular diameters with the callipers placed at the centre of the yolk sac (YS) wall; CRL was measured as the greatest length of the embryo in the anterior to posterior dimension; and HR was calculated from frozen M-mode images with electronic callipers by measuring the distance between two heart waves.

Intra- and interobserver reliability of measurements was tested on a random selection of 30 pregnancies at day 28 after ET. Each observer performed two measurements of GSD, YSD, CRL and HR on separate occasions and was unaware of others’ results. Written informed consent was obtained from all test patients before ultrasound scanning. The reference intervals were analysed according to the gestational days (GD) and GW. The GD can be deduced by adding 17 to the day of ET for cleavage stage embryos or adding 19 for blastocysts (Day 5 or Day 6), and the corresponding GW was obtained by dividing the GD by 7 [14]. The calculation method of fresh embryo and frozen embryo was the same.

### Statistical analysis

All statistical analyses were performed using SPSS software version 17.0 (SPSS, Inc., Chicago, IL, USA). Measurements are presented as the mean ± standard deviation (SD), and the enumerated data are expressed as numbers (percentages). The curve-fitting method was used to screen the optimal models for predicting GSD, CRL, YSD and HR based on GD and GW. We determined the optimal model based on the size of coefficient of determination (R^2^). The model with the largest R^2^ was ultimately selected as the best model. Additionally, the percentile method was used to calculate the 5th, 50th, and 95th percentiles for each time point. Scatter plots of GSD, CRL, YSD and HR compared with GD and GW were obtained. Correlation coefficients were calculated to analyse the intra- and interobserver reliability. A *p* value < 0.05 was considered significant.

## Results

From January 2010 to December 2016, 100,718 infertile patients obtained clinical pregnancies via IVF-ET in our hospital. After data exclusion, a total of 30,416 singleton pregnancies with normal outcomes were included in this study. The clinical characteristics of the study population were shown as Table 1. The measurements of GSD, CRL, YSD, and HR showed significant intra- and inter-observer correlations (*p* < 0.001).

**Table 1:** Clinical characteristics of the study population

Parameter	Value
Patients (n)	30,416
Age (years)	30.4 ± 4.4
BMI (kg/m^2^)	21.8 ± 2.8
Infertility duration (years)	4.8 ± 3.4
Transfer cycle (n)	1.2 ± 0.6
Infertility type	
Primary	13,971 (45.9%)
Secondary	16,445 (54.1%)
Cause of infertility	
Male	2513 (8.3%)
Female	17,946 (59.0%)
Combined female and male factors	8859 (29.1%)
Unexplained	1098 (3.6%)
Insemination methods	
IVF	14,445 (47.5%)
ICSI	5723 (18.8%)
IVF/ICSI	10,213 (33.6%)
PGD	35 (0.1%)
Embryo type	
Fresh	20,168 (66.3%)
Frozen	10,248 (33.7%)
Mode of delivery	
Spontaneous delivery	8165 (26.8%)
Caesarean section	22,251 (73.2%)
Birth weight (g)	3350.0 ± 240.0
5th centile	2550.0
50th centile	3350.0
95th centile	4100.0

Data are presented as n (%) or the mean ± SD

*BMI* body mass index, *IVF* in vitro fertilization, *ICSI* intracytoplasmic sperm injection, IVF/ICSI refers to either IVF or ICSI performed, *PGD* preimplantation genetic diagnosis (refers to PGT-M)

### Gestational sac diameter

**Fig. 2:**
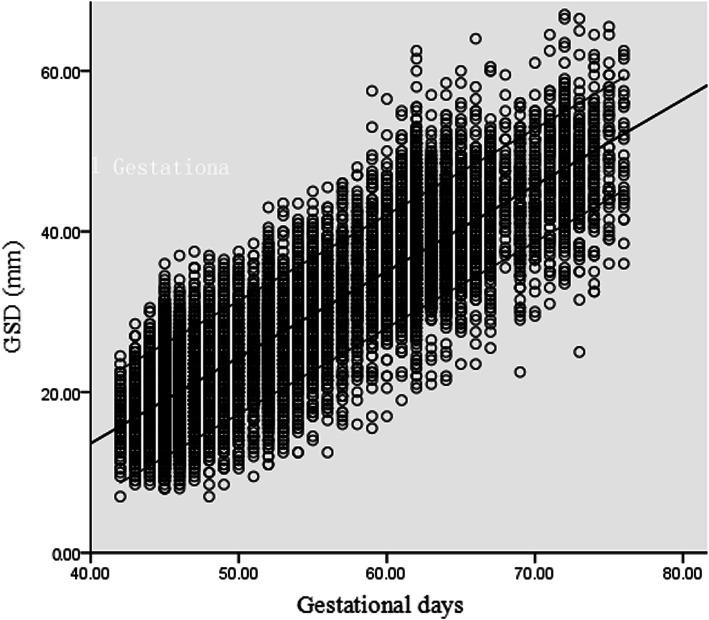
Scatter plots with the 5th, 50th, 95th percentiles of gestational sac diameter (GSD) against gestational days (GD)

There was a significant linear association between GSD and GA. The best fit models were as follows: GSD = -29.180 + 1.070 GD (R2 = 0.796, *P* < 0.001) and GSD = -29.180 + 7.492 GW (R2 = 0.796, *P* < 0.001). Figure 2 shows scatter plots with the 5th, 50th, 95th percentiles of GSD against GD.

### Crown–rump length

**Fig. 3:**
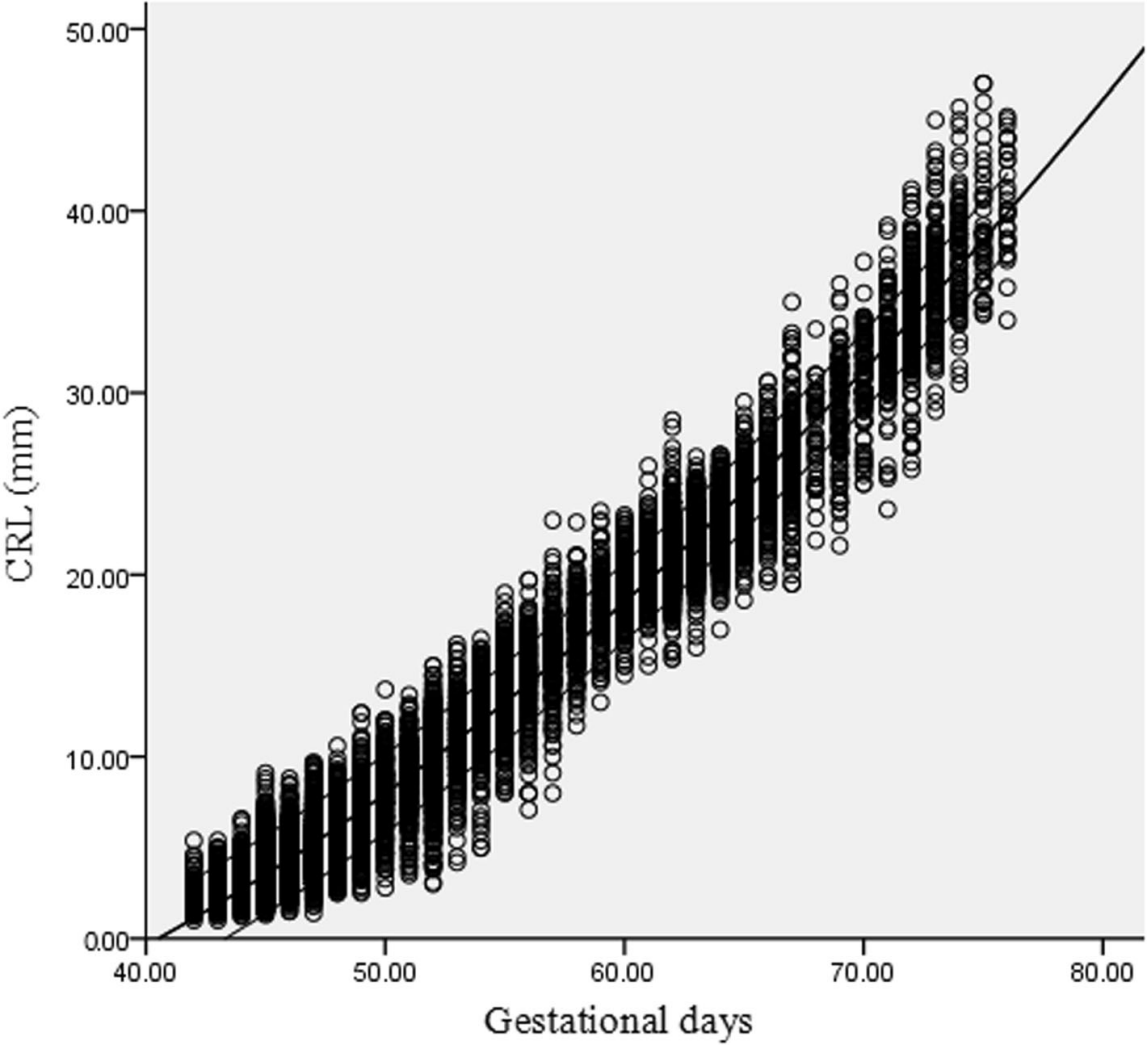
Scatter plots with the 5th, 50th, 95th percentiles of crown–rump length (CRL) against gestational days (GD)

There was a significant quadratic association between CRL and GA. The most appropriate fit models were as follows: CRL = − 11.960 - 0.147 GD + 0.011 GD^2^ (R^2^ = 0.976, *p* < 0.01) and CRL = − 11.960 - 1.028 GW + 0.535 GW^2^ (R^2^ = 0.976, *p* < 0.001). Figure 3 shows scatter plots with the 5th, 50th, 95th percentiles of CRL versus GD.

### Yolk sac diameter

**Fig. 4:**
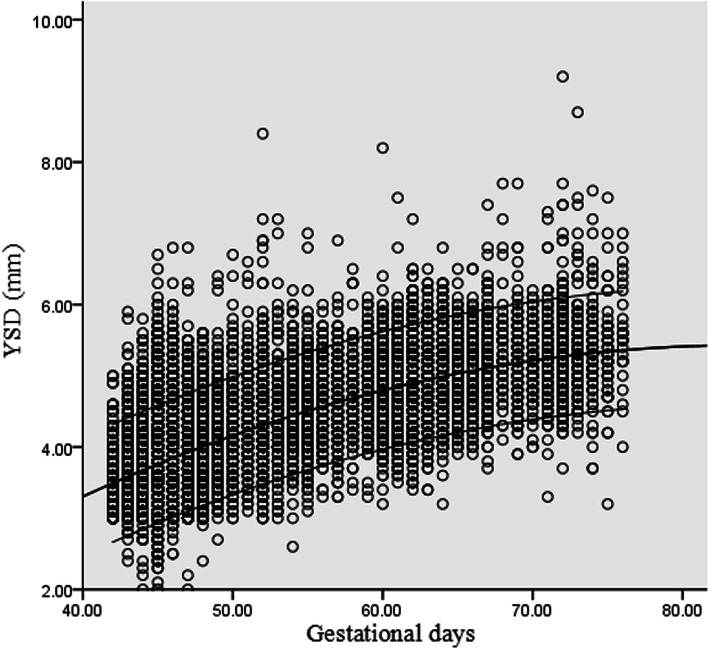
Scatter plots with the 5th, 50th, 95th percentiles of yolk sac diameter (YSD) against gestational days (GD)

A significant association between YSD and GA was found. The following quadratic models showed the most appropriate fit: YSD = − 2.304 + 0.184 GD - 0.011 GD^2^ (R^2^ = 0.500, *p* < 0.01), and YSD = − 2.304 + 1.288 GW - 0.054 GW^2^ (R^2^ = 0.500, *p* < 0.001). Scatter plots with the 5th, 50th, 95th percentiles of YSD against GD are presented in Fig. 4.

### Heart rate

**Fig. 5:**
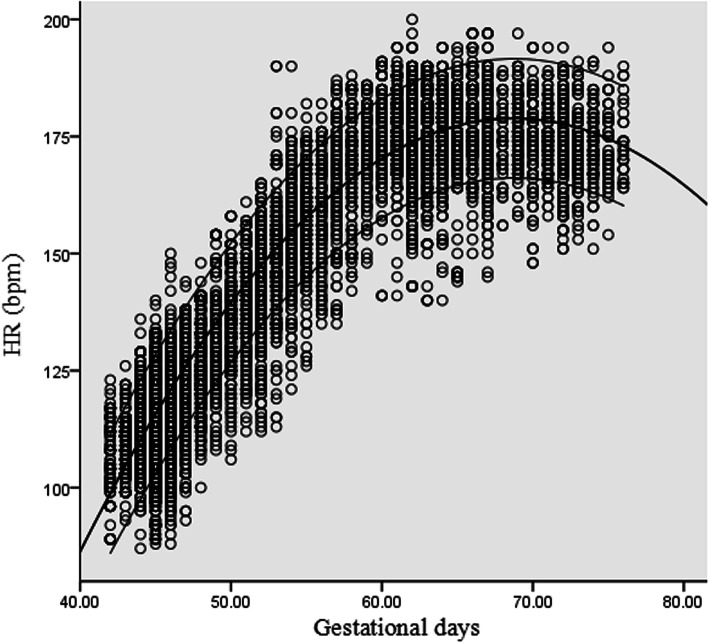
Scatter plots with the 5th, 50th, 95th percentiles of heart rate (HR) against gestational days (GD)

A significant association between HR and GA was found. The following quadratic models showed the best fit: HR = − 350.410 + 15.398 GD - 0.112 GD^2^ (R^2^ = 0.911, *p* < 0.001) and HR = − 350.410 + 107.788 GW - 5.488 GW^2^ (R^2^ = 0.911, *p* < 0.001). Scatter plots with the 5th, 50th, 95th percentiles of HR against GD are presented in Fig. 5.

Additionally, the details of the reference intervals for GSD, CRL, YSD and HR based on GD and GW are shown in Tables 2 and 3, respectively.

**Table 2:** Reference intervals for GSD, YSD, CRL and HR based on GD

Gestational days	n	GSD (mm)			CRL (mm)			YSD (mm)			HR (bpm)		
		5th	50th	95th	5th	50th	95th	5th	50th	95th	5th	50th	95th
42	159	11.5	16.0	21.5	1.5	2.2	4.0	3.0	3.6	4.5	96.0	108.0	117.0
43	503	12.0	17.0	23.5	1.8	2.6	4.1	3.0	3.6	4.6	101.2	109.0	115.0
44	2077	13.5	18.0	24.0	2.0	3.0	4.3	3.0	3.6	4.6	104.0	112.0	121.0
45	12,687	14.0	19.0	25.0	2.4	3.4	4.9	3.1	3.7	4.6	103.0	117.0	128.0
46	2726	14.0	19.5	26.0	2.6	3.9	5.7	3.1	3.8	4.7	103.0	118.0	129.0
47	1472	14.5	21.0	28.0	3.0	4.8	7.2	3.1	3.9	4.9	112.0	122.0	135.0
48	648	14.5	21.5	29.0	3.4	5.6	8.1	3.2	4.0	5.0	114.0	124.0	140.0
49	389	13.8	22.5	31.8	3.9	6.7	9.5	3.3	4.1	5.0	120.0	132.0	148.0
50	366	15.0	23.5	33.5	4.7	8.0	10.8	3.4	4.2	5.3	122.0	135.0	150.0
51	326	15.9	24.5	32.8	5.4	8.9	11.8	3.6	4.3	5.3	121.0	140.0	154.0
52	760	17.0	26.0	35.5	6.1	10.1	12.6	3.6	4.3	5.3	128.1	147.0	158.0
53	321	16.6	26.0	35.0	8.0	10.8	13.7	3.6	4.4	5.5	134.0	149.0	168.6
54	328	18.5	28.0	37.5	9.0	12.4	15.2	3.9	4.5	5.4	136.9	154.0	169.0
55	479	21.0	29.0	37.5	10.2	13.6	16.4	3.8	4.5	5.4	145.0	159.0	173.0
56	269	20.5	30.5	39.5	10.5	14.6	17.2	3.9	4.6	5.4	142.5	162.0	174.0
57	280	21.5	31.5	41.5	11.9	15.8	19.0	3.8	4.6	5.5	148.0	167.0	181.9
58	199	23.0	33.0	42.5	13.9	17.1	20.1	4.0	4.6	5.5	149.0	170.0	185.0
59	230	24.5	34.0	45.7	15.1	18.3	21.0	4.0	4.8	5.7	160.0	174.0	185.0
60	321	28.1	35.5	44.5	16.7	19.1	21.6	4.0	4.7	5.7	162.2	174.0	188.0
61	563	29.5	36.5	46.5	18.0	20.2	22.4	4.0	4.8	5.6	167.0	176.0	188.0
62	2247	30.5	37.5	47.3	19.0	21.0	23.2	4.2	4.8	5.6	160.0	176.0	188.0
63	662	31.0	38.0	47.5	19.5	21.8	24.4	4.1	4.9	5.7	161.2	176.0	188.0
64	583	31.5	39.0	48.9	20.3	23.0	25.3	4.2	5.0	5.8	164.0	178.0	188.0
65	319	32.0	40.0	50.5	21.0	24.0	26.9	4.2	5.0	5.8	164.0	176.0	186.0
66	207	32.5	40.0	50.5	21.7	25.0	28.8	4.2	5.1	5.9	164.0	178.0	190.6
67	282	33.6	42.5	51.0	22.5	26.5	30.8	4.4	5.2	6.0	158.2	176.0	190.0
68	57	37.5	44.5	55.2	23.9	28.9	31.0	4.3	5.2	6.8	161.4	174.0	188.0
69	157	31.4	43.5	53.0	24.0	29.1	33.0	4.3	5.2	6.1	166.9	176.0	189.1
70	106	35.9	43.5	54.5	26.0	31.0	34.1	4.4	5.1	6.0	156.4	176.0	186.7
71	121	35.1	46.0	56.4	27.9	32.3	36.4	4.3	5.2	6.2	164.0	175.0	188.0
72	255	38.4	47.0	57.0	29.2	34.3	38.4	4.4	5.2	6.2	158.0	174.0	186.0
73	132	37.0	46.0	56.2	31.6	36.4	41.7	4.5	5.4	6.7	159.7	174.0	186.7
74	104	37.0	48.0	60.3	33.1	38.0	42.9	4.2	5.3	6.5	160.0	173.5	185.0
75	51	39.0	49.0	62.4	34.5	38.3	46.4	4.2	5.3	6.9	162.6	171.0	186.2
76	30	37.4	49.0	62.2	35.0	40.1	45.1	4.3	5.5	6.9	164.0	172.0	188.9

**Table 3:** Reference intervals for GSD, YSD, CRL and HR based on GW

Gestational weeks	n	GSD (mm)			CRL (mm)			YSD (mm)			HR (bpm)		
		5th	50th	95th	5th	50th	95th	5th	50th	95th	5th	50th	95th
6	20,272	14.0	19.0	25.5	2.3	3.5	5.7	3.1	3.7	4.7	103.0	117.0	128.0
7	2969	16.5	26.0	35.5	5.3	10.1	14.8	3.5	4.3	5.3	124.0	146.0	164.5
8	4109	26.5	36.0	46.0	14.4	20.2	22.8	4.1	4.8	5.6	158.0	174.0	186.0
9	2267	31.5	40.0	50.0	20.2	23.4	29.8	4.2	5.0	5.8	162.4	176.0	188.0
10	799	37.0	46.5	57.5	29.0	34.5	41.2	4.4	5.3	6.4	160.0	174.0	186.0

## Discussion

In this study, we constructed reference intervals for GSD, YSD, CRL, and HR at 6–10 GW for an IVF population with a large sample of Chinese women. The optimal models for predicting GSD, CRL, YSD and HR based on GA were also presented.

In this study, a high proportion of CS is noted in Table 1. This high proportion of CS may be due to the high CS rate in China, which was estimated to be approximately 50% of births [11, 17]. However, the CS rate was as high as 73.2% in this study. The babies were conceived via IVF, and the implementation of the two-child policy in China has led to an increase in the number of elderly maternal pregnancies; over half of elderly mothers underwent CS for their first delivery; these factors might have contributed to the high CS rate in the IVF population [11].

Optimal models for predicting GSD, CRL, YSD and HR based on GA were established and showed that GSD linearly increases with GA. CRL, YSD, and HR had significant quadratic associations with GA. These models can be conveniently used in clinical practice to calculate the corresponding values of GSD, CRL, YSD and HR according to GA. However, the YSD models showed relatively lower R^2^ (0.500 for both GD and GW) than the other models, suggesting that the prediction models can only explain 50% of the changes in YSD; thus, in addition to GA, there are other factors to be explored.

The reference intervals for GSD, YSD, HR and CRL at 6–10 GW were constructed from a large sample in this study. This data can provide clinicians a reliable reference to analyse the development of early embryos after IVF-ET and facilitate monitoring of pregnancy outcomes at an early stage. GSD, YSD and CRL were found to gradually increase from 6 to 10 GW. However, HR increased from 6 GW, reaching a peak at 9 GW (176.0 bpm) and decreasing from there. This trend in HR was consistent with the results of previous studies [18, 19] and may be due to the development of the embryonic heart and its conductive system [20].

For comparison with previous studies, we performed a literature search of PubMed, and representative literature is listed in Table 4 [2, 5, 7, 21–26]. Most previous studies were conducted between the 1990s and 2000s and had small sample sizes of subjects with spontaneous conception or a mixed population. The most obvious difference between our study and previous studies was the CRL at early GA. In the studies by Grisolia et al. [22] and McLennan et al. [26], the CRL at day 45 was 7 mm; however, the CRL was 3.4 mm in our study. Both these studies used dating models among spontaneous conception or mixed populations to calculate GA according to CRL. Some researchers have suggested that the use of assisted reproduction data can improve dating accuracy; however, the accuracy is limited before 7 GW and is equivocal for menstrual dating beyond that GA [26], which may partly explain the considerable differences in CRL at day 45 between our study and previous studies. Additionally, CRL has been reported to overestimate gestation [27], and using CRL to determine GA has been reported to be less accurate than GA estimated by a certain LMP or day of oocyte retrieval in early pregnancy [28]; therefore, the CRL corresponding to the calculated GA is longer than the CRL of the same GA in IVF populations.

**Table 4:** Reference values for GSD, YSD, CRL and HR in previous studies and the present study

Author	Studied population	Inclusion criteria	Scanning method	Gestational days 45 55 65											
				GSD mm	CRL mm	YSD mm	HR bpm	GSD mm	CRL mm	YSD mm	HR bpm	GSD mm	CRL mm	YSD mm	HR bpm
**Robinson and Fleming, 1975** [7] **(*n*** **= 334)**	**Spontaneous**	**No information about outcome**	**TA**	**–**	**6.1**	**–**	**–**	**–**	**13.8**	**–**	**–**	**–**	**24.2**	**–**	**–**
**Lindsay et al.,** [21] **1992 (*n*** **= 327)**	**Spontaneous**	**Pregnancy continued ≥ 27 weeks**	**TV**	**–**	**–**	**2.7**	**–**	**–**	**–**	**3.1**	**–**	**–**	**–**	**3.5**	**–**
**Grisolia et al.,** [22] **1993 (*n*** **= 248)**	**Spontaneous**	**Normal live birth**	**TV/TA**	**14.0**	**7.0**	**–**	**–**	**26.0**	**15.0**	**–**	**–**	**38.0**	**25.0**	**–**	**–**
**Britten et al.,** [23] **1994 (*n*** **= 361)**	**Assisted reproduction**	**Normal live birth**	**TV**	**–**	**–**	**–**	**123.3**	**–**	**–**	**–**	**163.0**	**–**	**–**	**–**	**–**
**Yapar et al.,** [24] **1995 (*n*** **= 1331)**	**No information**	**No information about outcome**	**TV**	**–**	**–**	**–**	**128.2**	**–**	**–**	**–**	**161.6**	**–**	**–**	**–**	**175.1**
**Coulam et al.** [25] **1996 (n = 361)**	**Assisted reproduction**	**Normal live birth**	**TV**	**16.0**	**5.5**	**–**	**123.0**	**29.0**	**15.3**	**–**	**163.0**	**–**	**–**	**–**	**–**
**Tannirandorn et al.,** [2] **2000 (*n*** **= 547)**	**Spontaneous**	**Normal live birth**	**TV**	**–**	**–**	**–**	**147.4**	**–**	**–**	**–**	**163.9**	**–**	**–**	**–**	**172.7**
**McLennan et al.,** [26] **2008 (*n*** **= 396)**	**Mixed spontaneous + assisted**	**Pregnancy continued ≥ 20 weeks**	**TV/TA**	**–**	**7.0**	**–**	**–**	**–**	**15.0**	**–**	**–**	**–**	**26.0**	**–**	**–**
**Papaioannou et al.** [5] **2010 (*n*** **= 4698)**	**Mixed (spontaneous 97.9% + assisted 2.1%)**	**Normal live birth**	**TV**	**17.4**	**5.4**	**3.6**	**120.0**	**27.3**	**14.0**	**4.2**	**156.0**	**37.3**	**24.6**	**4.8**	**174.0**
**Our study 9** **(*n*** **= 30,416)**	**Assisted reproduction**	**Normal live birth**	**TV**	**19.0**	**3.4**	**3.7**	**117.0**	**29.0**	**13.6**	**4.5**	**159.0**	**40.0**	**24.0**	**5.0**	**176.0**

*GSD* gestational sac diamter, *CRL* crown rump length, *YSD* yolk sac diameter, *HR* heart rate, TA, transabdominal, *TV* transvaginal

The most popular formula for pregnancy dating originated from the study by Robinson and Fleming [7], and several studies proposing different dating equations have been reported since then. The use of different formulas can lead to discrepancies in GA estimation and corresponding differences in GSD, CRL, YSD and HR. In addition, different measurement methods may also lead to differences in ultrasound indicators. For example, when measuring YSD, some researchers prefer to place the calliper on the outside limits of the YS wall [29], while some place the calliper on the inner limits of the YS wall [30]. The measurements made in the study by Robinson and Fleming [7] were measured transabdominally, which might not be the same as measurements obtained transvaginally. Furthermore, the values in some articles were presented as means [23–25], while they were reported as medians in other studies [5, 22], which may also partly cause these differences.

Our study has several strengths. The large sample size allowed us to establish special reference intervals and construct optimal models for GSD, CRL, YSD and HR for IVF populations, which may be helpful for accurately analysing and monitoring the development of early pregnancy following IVF-ET. However, one potential weakness was that all the data were confined to one reproductive centre; although it is the largest centre in China, territorial limitations exist. Future studies with multi-centre samples are necessary to establish nationwide or worldwide references. Secondly, although the total sample was quite large, the patients were unevenly distributed. Most patients underwent their first ultrasound on day 28 after ET (45 GD, *n* = 12,687); however, much fewer patients underwent ultrasound on other days, particularly on later days. However, it is impractical to perform ultrasound for each patient every day to evenly distribute the sample. Therefore, future studies are needed to verify our reference intervals. Thirdly, to compare normal data with abnormal outcomes and try to understand whether the measurements may be somehow function as prognostic factors for abnormalities would be an interesting future work. Fourthly, since we collected the data retrospectively from the hospital database, some baseline data such as pharmacological treatments uses, parity, significant maternal diseases and smoking status were missing.

In addition, only fresh embryos, frozen embryos and days of transplantation were recorded for transplantation, but not blastocyst transplantation, so we were unable to further analyze the results of blastocyst transplantation. Previous studies have found lower uterine artery pulsatility index, proportion of small-for-gestational-age (SGA) [31] decreased risks of preterm \birth and low birth weight babies but a higher risk of large for GA babies as well as hypertensive disorders of pregnancies associated with pregnancies conceived from frozen embryos compared to fresh transfer [32]. While the difference between fresh and frozen embryos needs to be further confirmed by our follow-up studies. Another potential weakness was that IVF pregnancy may not be biologically equivalent to spontaneous conception due to increased risks of obstetrics and perinatal complications were shown for IVF pregnancies [33–35]. Thus, whether references based on IVF population are suitable for natural conceptions needs further elucidation.

## Conclusions

In conclusion, this study involving a large number of normal pregnancies presented the reference intervals for GSD, CRL YSD and HR at 6–10 GW. These data can be used as reliable references for analysing the development of early embryos after IVF-ET and for monitoring pregnancy outcomes at early stages.

## Data Availability

The data analysed during this study are included in the tables in this published article. The datasets used during the current study are available from the corresponding author on reasonable request.

## Abbreviations

GA: Gestational age
IVF-ET: In vitro fertilization-embryo transfer
GSD: Gestational sac diameter
YSD: Yolk sac diameter
CRL: Crown–rump length
HR: Heart rate
GW: Gestational week
GD: Gestational day
LMP: Last menstrual period
hCG: Human chorionic gonadotropin
TVS: Transvaginal scan
YS: Yolk sac
SD: Standard deviation
BMI: Body mass index
CS: Caesarean section
ICSI: Intracytoplasmic sperm injection
PGD: Preimplantation genetic diagnosis
